# Extracellular vesicles from *Trichinella spiralis*: Proteomic analysis and protective immunity

**DOI:** 10.1371/journal.pntd.0010528

**Published:** 2022-06-23

**Authors:** Xin Gao, Yong Yang, Xiaolei Liu, Fengyan Xu, Yang Wang, Lei Liu, Yaming Yang, Mingyuan Liu, Xue Bai

**Affiliations:** 1 Key Laboratory of Zoonosis Research, Ministry of Education, Institute of Zoonosis, College of Veterinary Medicine, Jilin University, Changchun, China; 2 Yunnan Institute of Parasitic Diseases, Puer, Yunnan, China; 3 Jiangsu Co-innovation Center for Prevention and Control of Important Animal Infectious Diseases and Zoonoses, Yangzhou, China; Guru Angad Dev Veterinary and Animal Sciences University, INDIA

## Abstract

*Trichinella spiralis* (*T*. *spiralis*) derived extracellular vesicles (EVs) have been proposed to play a key role in regulating the host immune responses. In this study, we provided the first investigation of EVs proteomics released by *T*. *spiralis* muscle larvae (ML). *T*. *spiralis* ML EVs (*Ts*-ML-EVs) were successfully isolated and characterized by transmission electron microscopy (TEM) and western blotting. Using liquid chromatograph mass spectrometer (LC-MS/MS) analysis, we identified 753 proteins in the *Ts*-ML-EVs proteome and annotated by gene ontology (GO). These proteins were enriched in different categories by GO, kyoto encyclopedia of genes and genomes (KEGG) and domain analysis. GO enrichment analysis indicated association of protein deglutathionylation, lysosomal lumen and serine-type endopeptidase inhibitor activity with proteins which may be helpful during parasite-host interaction. Moreover, KEGG enrichment analysis revealed involvement of *Ts*-ML-EVs proteins in other glycan degradation, complement and coagulation cascades, proteasome and various metabolism pathways. In addition, BALB/c mice were immunized by subcutaneous injection of purified *Ts*-ML-EVs. *Ts*-ML-EVs group demonstrated a 23.4% reduction in adult worms and a 43.7% reduction in ML after parasite challenge. Cellular and humoral immune responses induced by *Ts*-ML-EVs were detected, including the levels of specific antibodies (IgG, IgM, IgE, IgG1 and IgG2a) as well as cytokines (IL-12, IFN-γ, IL-4 and IL-10) in serum. The results showed that *Ts*-ML-EVs could induce a Th1/Th2 mixed immune response with Th2 predominant. This study revealed a potential role of *Ts*-ML-EVs in *T*. *spiralis* biology, particularly in the interaction with host. This work provided a critical step to against *T*. *spiralis* infection based on *Ts*-ML-EVs.

## Author summary

Extracellular vesicles (EVs) play an important role in cell-cell communication. They can transport functional molecules to target tissues and cells. During parasite infection, EVs provide suitable diagnostic makers and vaccine antigens for as well as inducing host immune responses. In *Trichinella spiralis* (*T*. *spiralis*), we previous proved that *Ts*-ML-EVs exerted immunomodulatory effect that inhibited experimental colitis in mice. Based on these results, we tested the protein composition of *Ts*-ML-EVs and the immune protective effect of *Ts*-ML-EVs. Our study showed that *Ts*-ML-EVs contained a number of immune-related proteins and it could be potential vaccine antigen for preventing T. spiralis infection.

## Introduction

Trichinellosis is a zoonotic disease caused by the genus *Trichinella*, primarily *Trichinella spiralis* (*T*. *spiralis*) [1]. Trichinellosis is both a public health hazard and a significant economic burden on food safety [2]. The life cycle of *T*. *spiralis* comprises two generations within the same host. Host infections begin with the ingestion of *T*. *spiralis* muscle larvae (ML) within the muscle tissue of domestic or wild animals [3]. ML then penetrate the intestinal mucosa and develop into the adult worm. After delivery by female adult worms, newborn larvae (NBL) migrate to striated muscle cells [4]. The NBL subsequently develop into infective ML, which can live in the striated muscle for many years. In order to successfully invade and establish infection in the host, *T*. *spiralis* has evolved variety of complex immunomodulatory mechanisms at different stages of parasitic growth to evade or suppress the immune response [5]. Therefore, further research is needed to develop new prevention strategies against *T*. *spiralis*.

In recent years, extracellular vesicles (EVs) have been considered to be an important component of parasite immune escape strategies through the transport of immunomodulatory cargo molecules [6]. EVs seem to be the main way that parasite export proteins, and some even contain host homologous proteins [7]. It has been found that EVs released by several parasites can deliver bioactive molecules to host cells, thereby regulating host gene expression [8]. Selective packaging of proteins may allow the parasite to migrate and establish within the host [9]. EVs from *Fasciola hepatica* contain many established immunoregulatory proteins, including Sigma class glutathione transferase [10]. *Taenia pisiformis* cysticercus EVs contain 87 proteins, including proteins associated with EVs biogenesis, and these induce a Th2-type immune response in RAW264.7 macrophages [11]. Similarly, EVs signaling appears to be the main mechanism through which *Leishmania* transmits virulence factors to host cells [12]. The above studies provide evidence that parasite EVs is a mechanism by which parasites secrete proteins to the host, and EVs proteins are important mediators of biological processes such as adherence, immunomodulation and inflammation [13]. The study of EVs and their protein components is beneficial for the development of anti-parasitic vaccines, which will help to further explore how to control parasitic diseases.

It has been confirmed that many of these EVs functional proteins have the potential for immune protection against parasitic infection. C57BL/6 mice immunized with *Heligmosomoides polygyrus* EVs-alum produced high levels of IgG1, demonstrate a protective immunity against parasitic challenge, highlighting the important role of EVs functional proteins *in vivo* [14]. Evidence that the biological fate of EVs from parasites can be altered by anti-EVs antibodies is accumulating. Antibodies to *Heligmosomoides polygyrus* EVs alter the intracellular distribution of EVs, directing them enter the lysosomal pathway [14]. *Echinococcus granulosus* secretes EVs containing many functional proteins (including 14-3-3 proteins), and 14-3-3 protein could provide 97% protection against infection after parasite challenge in rodent [15]. Proteins of the tetraspanin family (TSPs) on the surface of *Opisthorchis viverrini* EVs have proven to be an effective vaccine in a hamster model of metacercariae infection [16]. In addition, antibodies to *Opisthorchis viverrini* tetraspanin block uptake of EVs and the secretion of IL-6 by cholangiocytes [17]. Therefore, it is important to identify EVs proteins for the development of vaccines against parasite infection.

*T*. *spiralis* ML EVs (*Ts*-ML-EVs) have previously been isolated and characterized in our laboratory. *Ts*-ML-EVs could significantly inhibit 2,4,6-trinitrobenzene sulfonic acid (TNBS) induced colitis in BALB/c mice by promoting Th2 immune responses and their small RNA composition is involved in the regulation of inflammation [18]. In addition, *Ts*-ML-EVs can affect the inflammatory development of DSS-induced colitis by inhibit M1 macrophage polarization, due to their immunomodulatory ability [19]. However, information on the protein composition of *Ts*-ML-EVs is limited.

In this study, we provided the first proteomic analysis of *Ts*-ML-EVs with the aim of describing the interaction of *Ts*-ML-EVs and host. We identified 753 proteins in *Ts*-ML-EVs. And we demonstrated that these proteins performed multiple functions by GO and KEGG analysis. Moreover, *Ts*-ML-EVs contained many vaccine candidate antigens thus we investigated the protective immunity afforded by *Ts*-ML-EVs against *T*. *spiralis* infection. We found that *Ts*-ML-EVs could induce Th2 dominated Th1/Th2 mixed responses by detecting antibodies and cytokines levels in serum of BALB/c mice. Our results help to understand the role of *Ts*-ML-EVs and the development of vaccines to prevent *T*. *spiralis* infection.

## Materials and methods

### Ethics statement

Animal research was conducted in accordance with the Administration of Affairs Concerning Experimental Animals guidelines in China. The experimental protocol was approved by the Institutional Animal Care and Use Committee of Jilin University (20170318).

### Experimental animals and preparation of *Trichinella spiralis* muscle larvae

Female BALB/c mice (19–21 g) and female Wistar rats (170–200 g) were obtained from the Experimental Animal Centre of College of Basic Medical Sciences. Experimental animals (eight per cage) were subjected to controlled laboratory conditions (temperature, 18–26°C; 12 h of light/ 12 h of darkness from 9.00 p.m. to 9.00 a.m.). Animals were provided water and food ad libitum, and the sawdust in the cage was changed every 2 days.

After oral infection of Wistar rats with a suspension containing 3500 infectious muscle larvae (ML), *T*. *spiralis* (ISS534) ML were harvested [20]. Briefly, infected rats were anesthetized with chloral hydrate and sacrificed by cervical dislocation at day 35 after infection. All procedures were carefully designed to minimize pain. The muscles of infected rats were homogenized, and then added to a pepsin solution (1% pepsin; 1% HCl) in a ratio of 1:30. The homogenized muscle suspension was then incubated at 37°C for 2 h for digestion. Finally, the suspension was filtered through 600 μm meshes and repeatedly sedimented until clear. The presence of ML in the sediments was then confirmed using a microscope.

### Isolation of extracellular vesicles from *Trichinella spiralis* muscle larvae

The collected *T*. *spiralis* ML were washed several times, and then cultured in pre-heated RPMI-1640 medium. ML were incubated at 37°C in 5% CO2. After 12 h, the culture medium containing ML excretory secretion products (*Ts*-ML-ES) were centrifugated at 800×g for 15 min, followed by centrifugation at 5000×g for 15 min to remove ML [21]. The culture supernatant was then filtered through a 0.22 μm filter to remove debris, and the filtrate was concentrated using ultracentrifugation filters (Merck Millipore, USA) to a final volume of 150 mL. Finally, *T*. *spiralis* extracellular vesicles (*Ts*-ML-EVs) were harvested by ultracentrifugation at 120,000×g for 2 h, as described in previous reports [18]. The concentration of *Ts*-ML-EVs was measured by BCA kit. The *Ts*-ML-EVs were then stored at -80°C for further analysis.

### Transmission electron microscopy

To characterize the *Ts*-ML-EVs in our sample, freshly isolated *Ts*-ML-EVs (10 μL) were directly absorbed onto a copper grid, fixed with 2% glutaraldehyde, and then stained with 2% phosphotungstic acid. The *Ts*-ML-EVs samples were then immediately observed using a HitachiH-7650 transmission electron microscope (Hitachi Limited, Japan). The diameter of *Ts*-ML-EVs was then measured using ImageJ software (1.41).

### Western blot analysis

*T*. *spiralis* ML were resuspended in cell lysis buffer and incubated at room temperature for 5 min. The lysate was then centrifuged for 15 min at 12,000 rpm to obtain total ML proteins. *Ts*-ML-EVs and ML protein samples (30 μg) were then separated by electrophoresis on 10% SDS-PAGE gel. The separated proteins were then transferred to a PVDF membrane (Millipore, USA) for western blot analysis. After a 2 h blocking step in 5% skim milk, Enolase was detected by incubation with polyclonal goat anti-enolase (1:200; Abcam, United Kingdom) overnight at 4°C, followed by incubation with donkey anti-goat IgG-horseradish peroxidase conjugate (1:50,000; Jackson ImmunoResearch, USA) at room temperature for 2 h. Visual detection of membranes was performed using the ECL Plus Western blotting detection system (GE Healthcare Buckinghamshire, UK).

### Proteomic analysis

The *Ts*-ML-EVs sample was sonicated using an ultrasonic processor (Scientz) in the presence of 1% protease inhibitor. The sample was then clarified by centrifugation at 12,000×g for 10 min at 4°C. The supernatant was collected and the protein concentration was determined using a BCA kit (according to the manufacturer’s instructions). Finally, 5 μg of *Ts*-ML-EVs protein was loaded on a 12% SDS-PAGE gel for electrophoresis. The separated proteins were then visualized by silver staining.

Samples from the gel were submitted to the PTM BIO Service for liquid chromatography/ mass spectroscopy (LC-MS/MS, Thermo Scientific, USA) analysis. Briefly, specific protein bands were digested with trypsin to obtain tryptic peptides for subsequent analysis. The tryptic digests were then separated and individual peptides were identified by LC-MS/MS using a Q Exactive mass spectrometer coupled to an EASY-nLC 1200 UPLC system. The samples were ionized by electrospray (Thermo Scientific, USA), and the data were analyzed using the Maxquant search engine (v.1.5.2.8). The sequence dataset used for the analysis of the LC-MS/MS analysis was Trichinella _ spiralis (18572 sequences) from UniProt database.

The *Ts*-ML-EVs proteins identified were then analyzed by Gene Ontology (GO) classification using the UniProt-GOA database (http://www.ebi.ac.uk/GOA/). The Kyoto Encyclopedia of genes and Genomes (KEGG) database was also used to annotate protein pathways. In addition, the *Ts*-ML-EVs proteins identified were searched against the STRING database (version 11.0) for protein-protein interactions. All interactions with a confidence score > 0.7 (high confidence) were included. The STRING interaction network was then visualized using the R package “networkD3”.

### Immunization and challenge protocol

For immunization, eight mice in 3 groups were subcutaneous injected with PBS, PBS+IMS1313 (SEPPIC), and *Ts*-ML-EVs (s. c.) (with IMS1313) on day 0, day 14 and day 28. IMS1313 was used as an adjuvant. All samples contained 30 μg *Ts*-ML-EVs in 100 μL of PBS and 100μL IMS1313. Two weeks after the last immunization, the 3 groups of animals were challenged with 300 ML [22]. All experiments were repeated three times. The numbers of adult worms (4 mice) and ML (4 mice) were determined on day 6 and day 35. The mice were anesthetized with isoflurane before blood collection. Capillary needle was inserted into the inferior orbital venous plexus of mice. After 200–300 μL blood was collected, the blood collection needle was pulled out and the mouse eye was pressed with a clean cotton ball to stop bleeding. Put the mice in a separate cage and pay attention to their recovery and physical condition. Blood samples allowed to clot for 2 h at room temperature before centrifugation at 1000×*g* for 15 min. The serum was then stored at -20°C.

### Analysis of antibody response and cytokines expression by ELISA

Serum samples were collected to analyze cytokines (IL-4, IL-10, IL-12, and IFN-γ) using ELISA kits (Cusabio, CN) according to the manufacturer’s instructions. The expression of cytokines was inferred from the standard curve provided by the ELISA kits. Indirect ELISA was used to detect the *Ts*-ML-EVs specific IgG, IgG1, IgG2a, IgM and IgE antibody levels. ELISA plates were coated with 1μg/mL *Ts*-ML-EVs at 4°C overnight. After blocking with 5% skimmed milk for 1h at 37°C, serum was added in serial dilutions to ELISA plates. Antibody binding was detected using horseradish peroxidase (HRP)-conjugated goat anti-mouse IgG, IgG1, IgG2a, IgM and IgE (Southern Biotech) and read at 450 nm.

### Statistical analysis

All results are expressed as mean ± SD. Statistical analysis was performed using GraphPad Prism 8 for Windows. One-way analysis of variance (ANOVA) was used to compare statistical differences between the different conditions. And Dunnett’s multiple comparison was used to describe the statistical analysis (compared with the PBS group). *P* values are expressed as *P < 0.05, **P < 0.01, ***P < 0.001 and ****P < 0.0001 (compared with the PBS group).

## Results

### Proteomics analysis of *Ts*-ML-EVs

**Fig 1:**
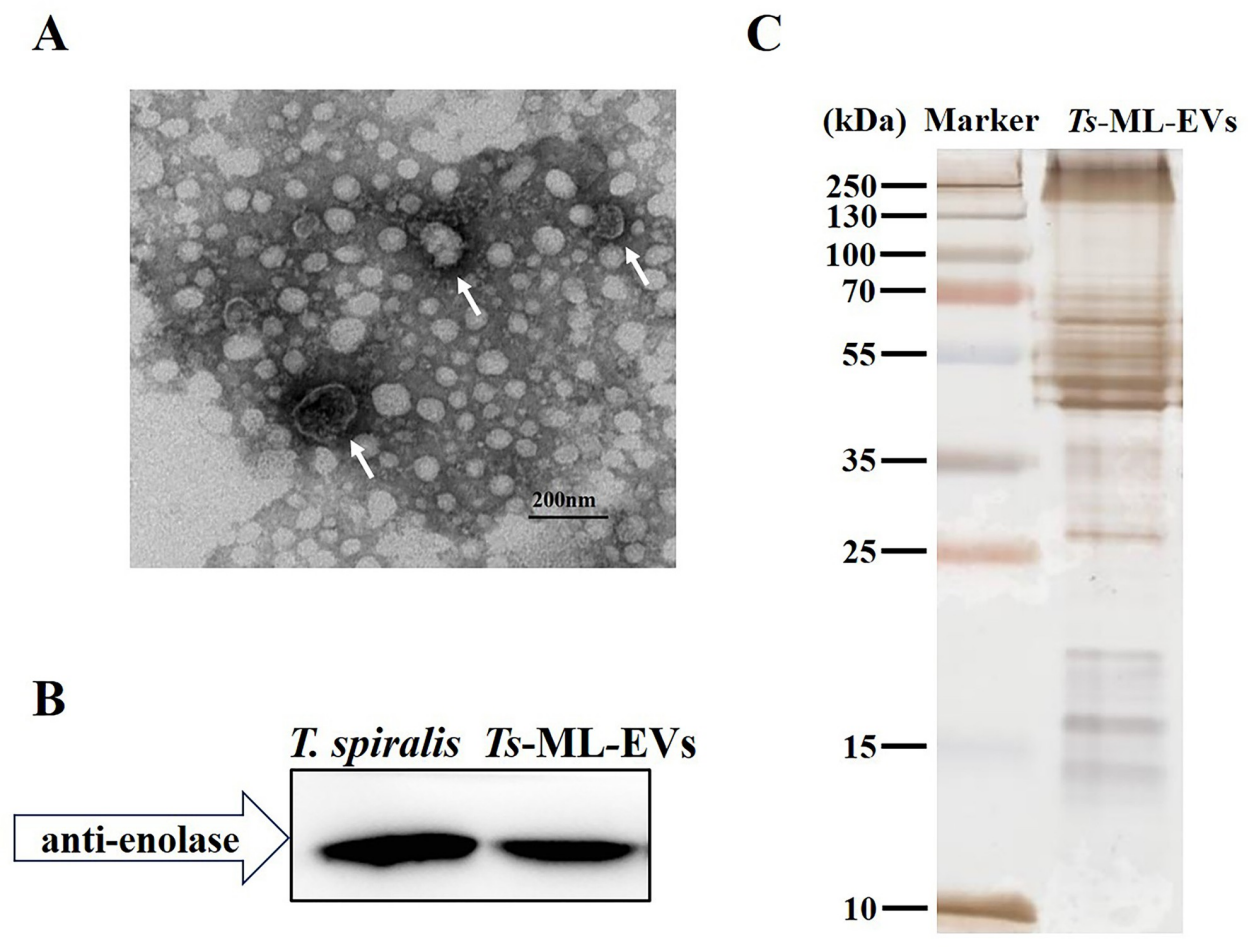
Characterization and protein analysis of *Ts*-ML-EVs. (A) Morphological characterization of *Ts*-ML-EVs by TEM. Arrows indicate isolated *Ts*-ML-EVs. (B) Western blot analysis showing the marker protein of *Ts*-ML-EVs. (C) SDS-PAGE analyses of purified *Ts*-ML-EVs using silver-staining.

In order to obtain *Ts*-ML-EVs, vesicles were isolated from RPMI-1640 medium as previously described [19]. Transmission electron microcopy (TEM) analysis showed that the vesicles were round with bilayer (Fig 1A) [18]. Western blot analysis revealed that the *Ts*-ML-EVs express the EVs marker enolase (Fig 1B). Together, these observations provide evidence for the successful isolation of *Ts*-ML-EVs. To elucidate the constituents of the *Ts*-ML-EVs proteome, *Ts*-ML-EVs total protein was analysed by SDS-PAGE and silver staining. The result showed that proteins contained in EVs, mainly distributed in the molecular weight of 45-70kDa (Fig 1C). To further determine the protein composition of *Ts*-ML-EVs, proteomic analysis of isolated bands was performed using LC-MS/MS. Proteomic results showed that 753 proteins were identified in EVs secreted in *T*. *spiralis*. The top 50 enriched *Ts*-ML-EVs proteins were mainly including translation regulatory proteins, nuclear regulatory proteins, signal transduction proteins, cytoskeletal proteins, serpins, enzymes, secreted proteins, transporters, immunity related proteins, protease inhibitors, metabolism related proteins, metabolic enzymes and other proteins (S1 Table).

### Functional annotation and cellular localization of EVs proteins in *T*. *spiralis*

**Fig 2:**
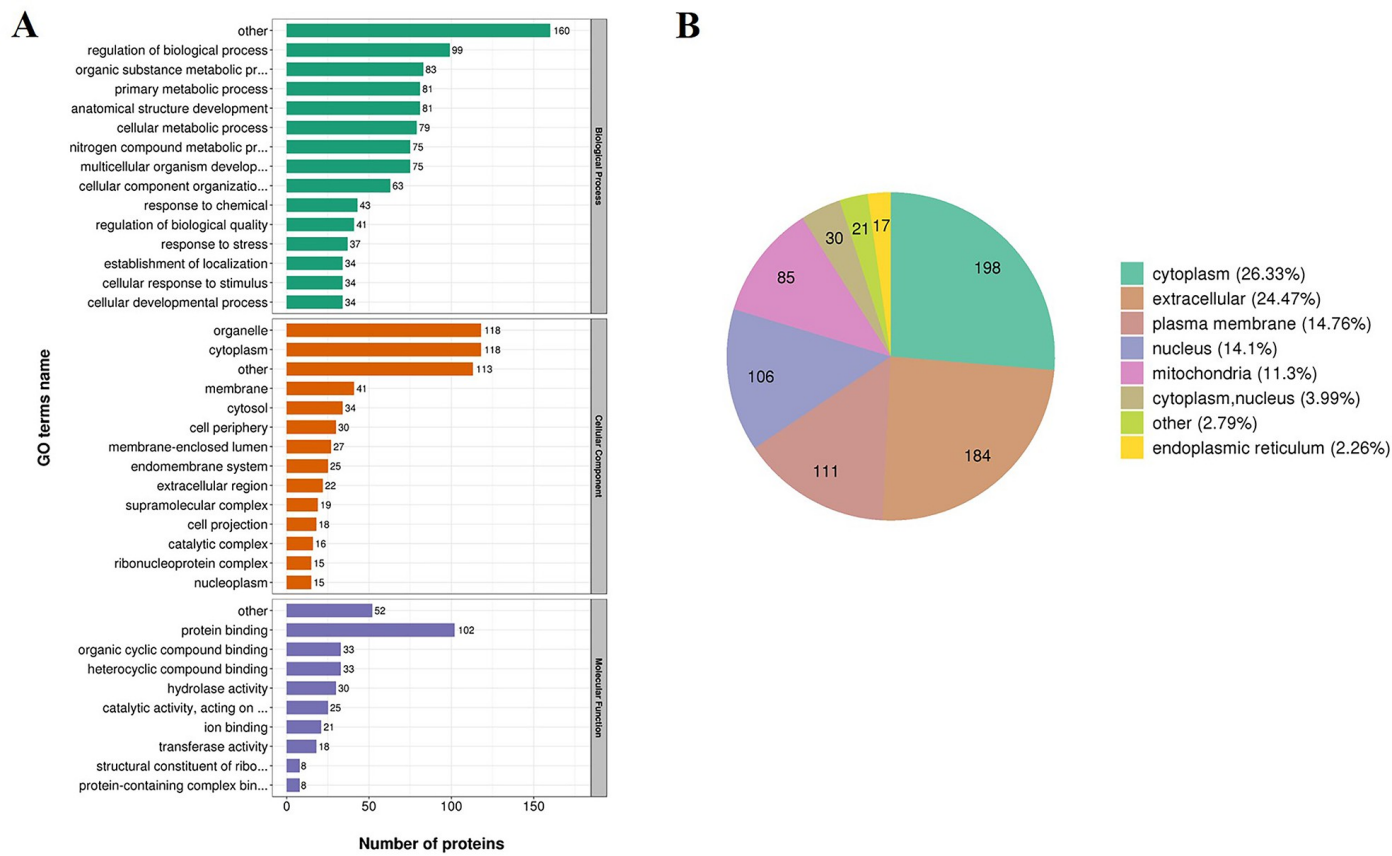
Bioinformatic analyses of proteins in *Ts*-ML-EVs. (A) Gene ontology (GO) annotation of all 753 proteins in *Ts*-ML-EVs. (B) Protein subcellular localization mapping of proteins.

We annotated proteins of *Ts*-ML-EVs with Gene ontology (GO) analysis. The result showed that 202 proteins were associated with “Biological process”, 182 proteins were associated with “Cellular component”, and 170 proteins were associated with “Molecular function” (Fig 2A). Because regulation of *Ts*-ML-EVs protein distribution is essential for intracellular physiological homeostasis, we next examined the subcellular distribution of *Ts*-ML-EVs proteins, and classified them according to their distribution. *Ts*-ML-EVs proteins were mostly distributed in cytoplasm (26.33%) or extracellular (24.47%) locations (Fig 2B). However, *Ts*-ML-EVs proteins were also distributed in the plasma membrane (14.76%), nucleus (14.1%), and mitochondria (11.3%). Subcellular localization analysis showed that *Ts*-ML-EVs proteins were distributed in different cell compartments and may involve in a variety of biological functions.

### Enrichment analysis of *Ts*-ML-EVs proteins

**Fig 3:**
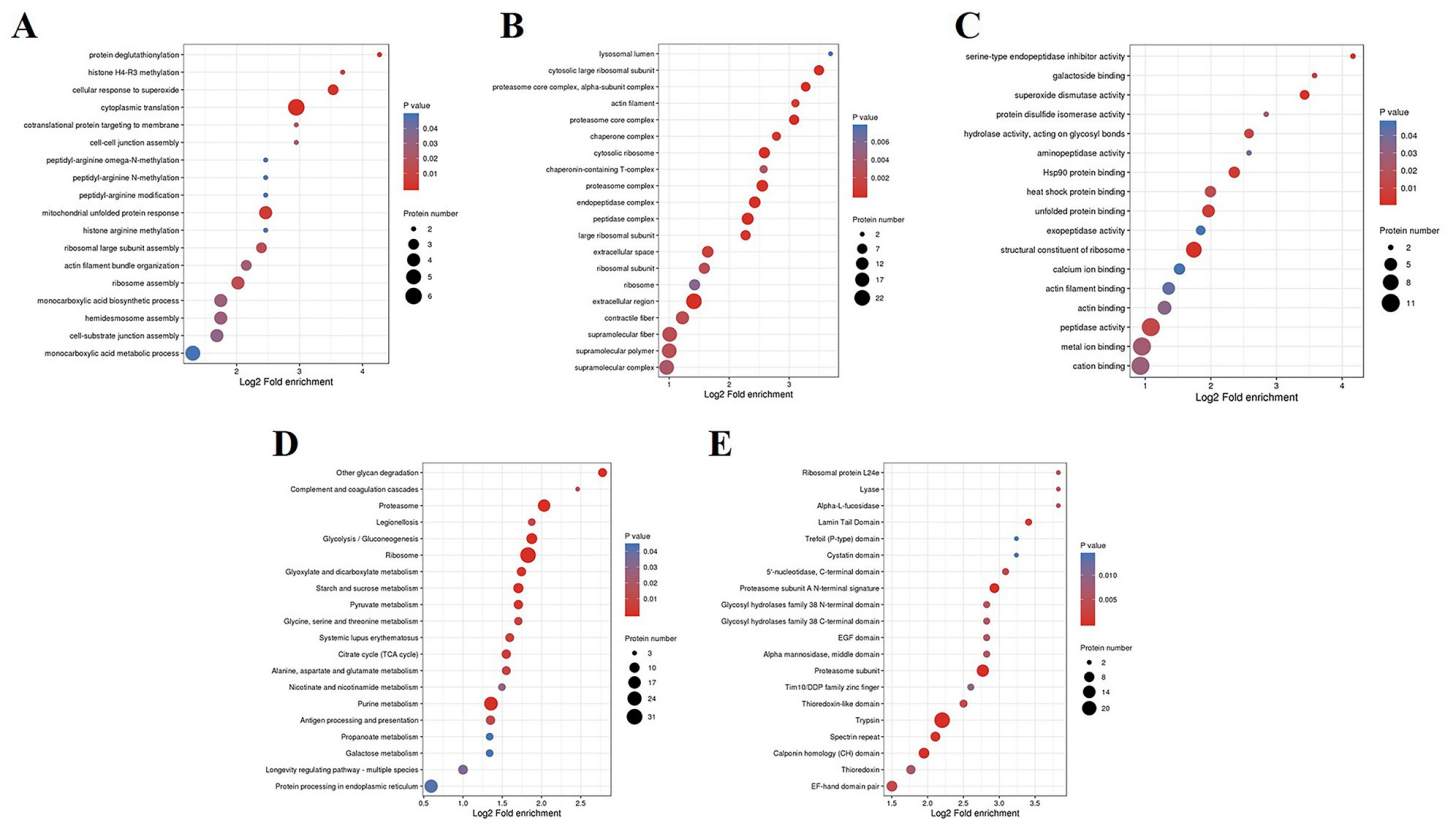
Enrichment analyses of the proteins derived from *Ts*-ML-EVs. Enrichment analyses by “Biological process” (A), “Cell component” (B), “Molecular function” (C), KEGG pathway (D), and (E), protein domain.

A total of 753 *Ts*-ML-EVs proteins were categorised according to gene ontology (GO) and KEGG pathway, and protein domain. The top 20 classifications showing the most significant enrichment scores were screened further. In the case of “Biological process”, *Ts*-ML-EVs proteins were most significantly enriched in the protein deglutathionylation, histone H4-R3 methylation, cellular response to superoxide, cytoplasmic translation, and cotranslational protein targeting to membrane categories (Fig 3A). GO enrichment of “Cellular component” revealed that *Ts*-ML-EVs proteins were most significantly enriched in lysosomal lumen, cytosolic large ribosomal subunit, proteasome core complex, alpha-subunit complex, actin filament and proteasome core complex categories (Fig 3B). In the case of “Molecular function”, *Ts*-ML-EVs proteins were most significantly enriched in serine-type endopeptidase inhibitor activity, galactoside binding, superoxide dismutase activity, protein disulphide isomerase activity and hydrolase activity, acting on glycosyl bonds categories (Fig 3C). To identity the associated pathways, we performed KEGG analysis, and found that the identified proteins were enriched in 347 pathways. Further analysis revealed that *Ts*-ML-EVs proteins were significantly enriched in other glycan degradation, complement and coagulation cascades, proteasome and various metabolism pathways (Fig 3D). Protein domains are evolutionary conserved amino acid sequence (25–500 amino acids in length) found in different protein molecules. These protein domains are usually functionally and structurally similar. To explore the conservation of *Ts*-ML-EVs proteins, we first examined the enrichment of protein domains. The top 3 enriched domains were “Ribosomal protein L24e”, “Lyase”, and “Alpha-L-fucosidase” domains (Fig 3E). Besides, *Ts*-ML-EVs protein domains were also enriched in “Lamin Tail Domain”, “Trefoil (P-type) domain” and “Cystatin domain”.

### *Ts*-ML-EVs stimulated protective immunity against *T*. *spiralis*

**Fig 4:**
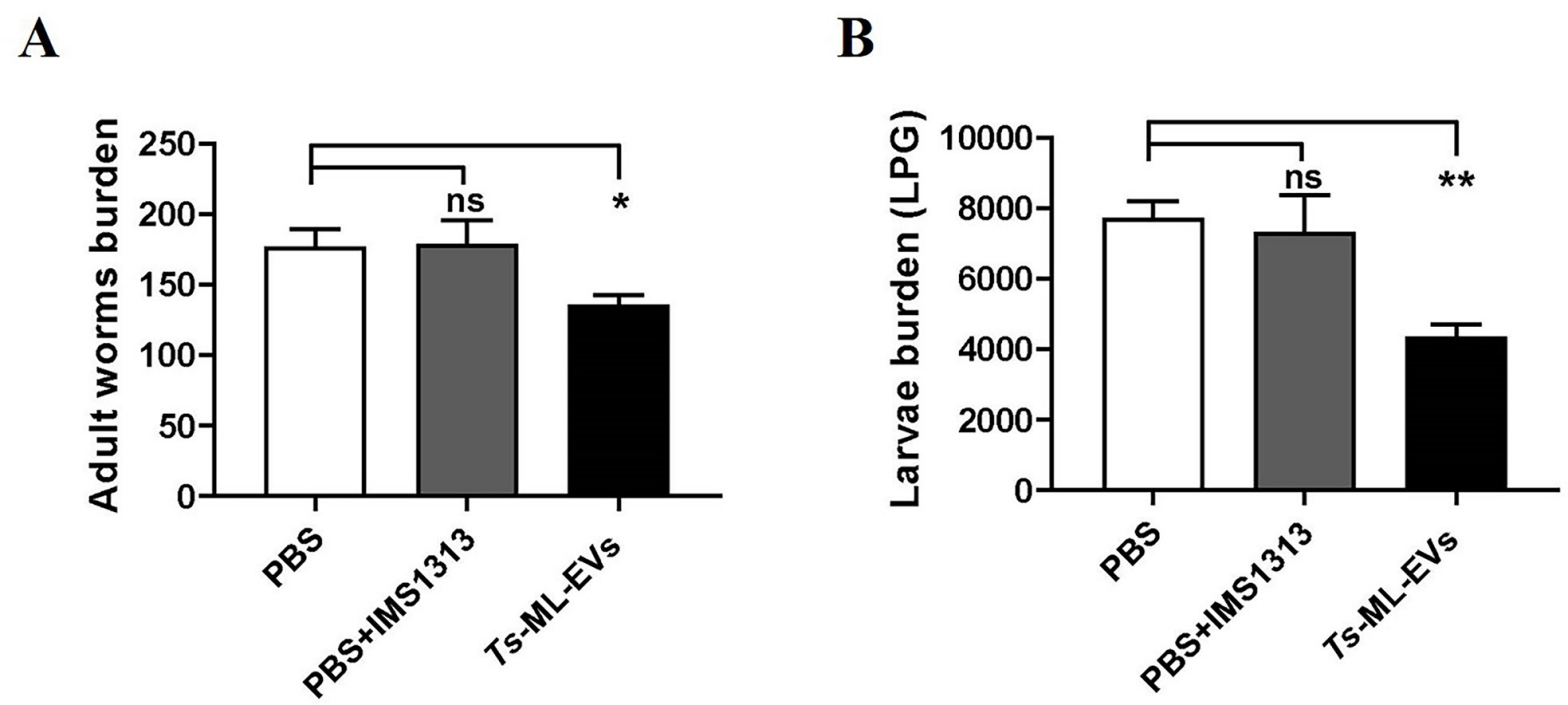
Immunization with *Ts*-ML-EVs elicited a delay in the course of the infection and increased the survival rate. (A) Worm burden from 4 vaccinated mice with mean and SD bar shown. *P<0.05, **P<0.01, ***P<0.001. (B) The LPG from 4 vaccinated mice with mean and SD bar shown. Data were evaluated by Dunnett’s multiple comparison poet hoc test. All data are pooled from three independent experiments. *P<0.05, **P<0.01, ***P<0.001.

To determine whether *Ts*-ML-EVs can induce a protective immune response *in vivo*, BALB/c mice were immunized with purified *Ts*-ML-EVs in a formulation with adjuvant (given on days 0, 14, and 28) and subsequent *T*. *spiralis* infection. On day 6, intestinal adult worm burden of *Ts*-ML-EVs group was reduced by 23.4% (*P*<0.05) after infection (Fig 4A). And on day 35, ML burden of *Ts*-ML-EVs group was reduced by 43.7% (*P*<0.01) after infection (Fig 4B). These results provide evidence that vaccination of mice with *Ts*-ML-EVs can elicit partial immune protection against *T*. *spiralis* larval infection.

### *Ts*-ML-EVs induced systemic immune response against *T*. *spiralis* challenge

**Fig 5:**
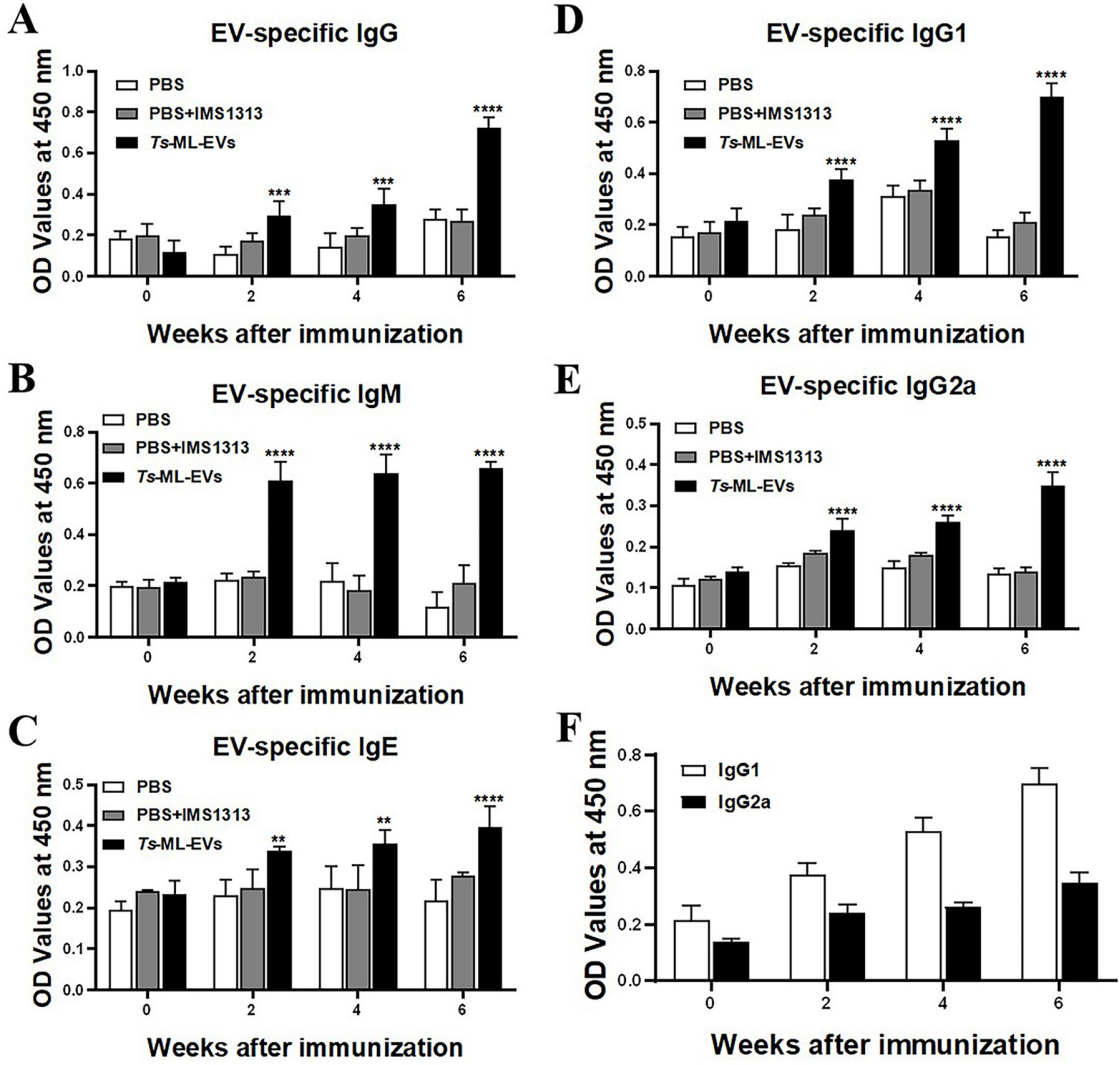
EV-sepecific antibodies detected from sera of immunized BALB/c mice. IgG (A), IgM (B), IgE (C), IgG1 (D) and IgG2a (E) antibodies against *Ts*-ML-EVs produced were analyzed by ELISA using sera (n = 8/group) from each group. (F) IgG subclass responses of mice immunized with *Ts*-ML-EVs were detected at different time points. Results represent the mean absorbance measured at 450nm from each group. Data were evaluated by Dunnett’s multiple comparison poet hoc test. All data are pooled from three independent experiments. *P<0.05, **P<0.01, ***P<0.001, ****P<0.0001.

Elevated serum antibodies are commonly used as anti-parasitic responses in infected mice. Serum from all 3 group was collected on day 0. PBS, PBS+IMS1313, and *Ts*-ML-EVs+IMS1313 were then administered subcutaneously three times. Serum was collected before each injection to track the production of specific antibodies against *Ts*-ML-EVs. After two weeks, IgG increased in the *Ts*-ML-EVs group (compared to the PBS group). The increase observed after the four weeks was even more significant, confirming that *Ts*-ML-EVs can induce an immune response in host. There was no significant difference in IgG level between PBS group and PBS+IMS1313 group (Fig 5A). From two weeks after the immunization with *Ts*-ML-EVs, IgM increased significantly and remained high throughout the immunization period (Fig 5B). In addition, IgE levels were specifically elevated in mice immunized with *Ts*-ML-EVs (Fig 5C). IgG1 antibody levels were significantly higher in the *Ts*-ML-EVs group compared to the PBS group (Fig 5D). High levels of anti-parasitic IgG2a were also detected in the *Ts*-ML-EVs group, which may indicate that *Ts*-ML-EVs group mice produced a mixed Th1/Th2 response (Fig 5E). In addition, levels of IgG1 were higher than IgG2a, confirming that *Ts*-ML-EVs mainly stimulated Th2 immunity (Fig 5F).

**Fig 6:**
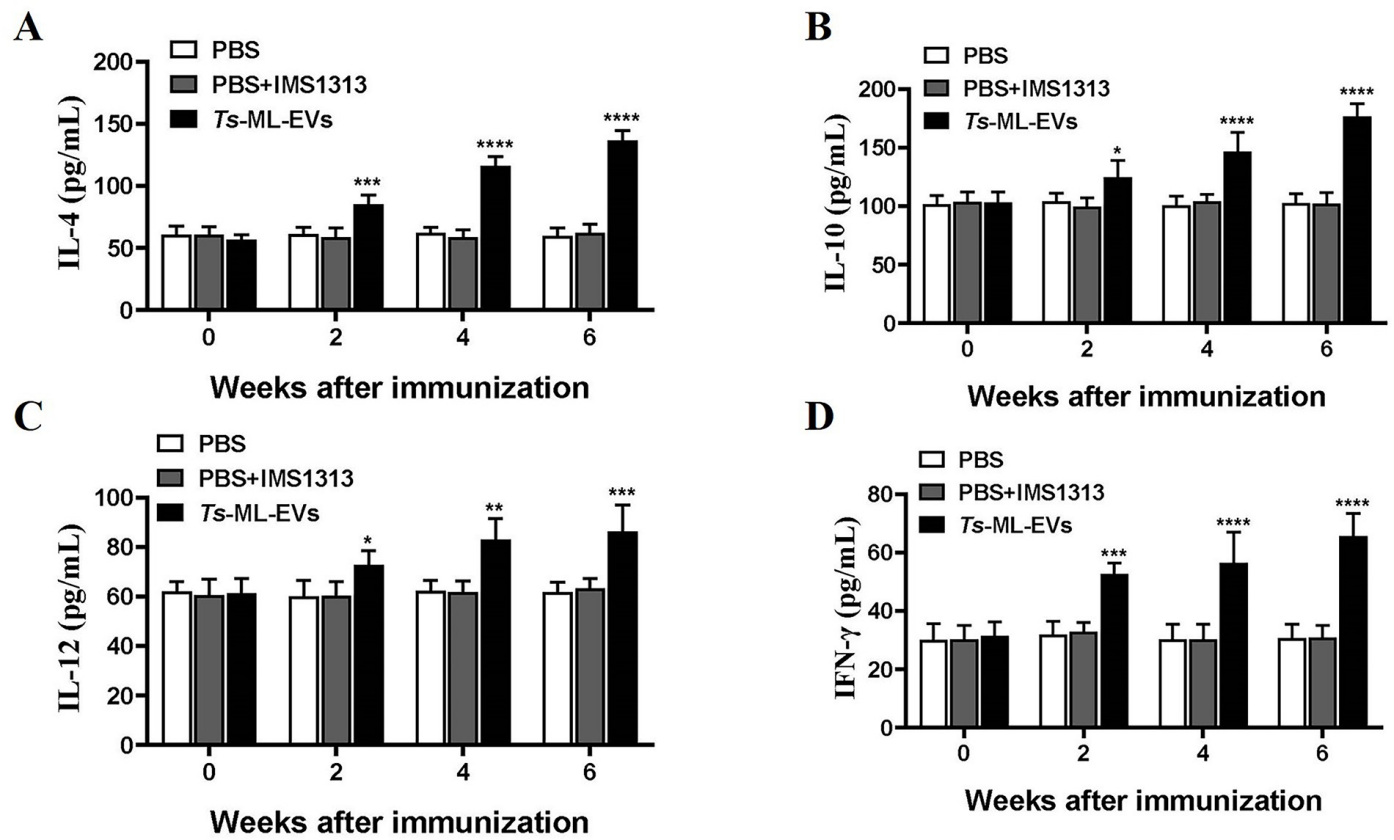
Vaccination results increased expression of cytokines in serum. Expression levels of IL-4 (A), IL-10 (B), IL-12 (C) and IFN-γ (D) stimulated with *Ts*-ML-EVs were determined by ELISA. Results represent the mean absorbance measured at 450 nm from each group. Data were evaluated by Dunnett’s multiple comparison poet hoc test. All data are pooled from three independent experiments. *P<0.05, **P<0.01, ***P<0.001, ****P<0.0001.

Analysis of cytokine expression is essential to determine the outcome of parasitic infection and to understand the mechanisms of parasite rejection or host protection. Thus, we examined the modulatory effects of *Ts*-ML-EVs on cytokines. Serum samples were collected at 0, 2, 4 and 6 weeks after immunization with *Ts*-ML-EVs. Th2-type cytokine (IL-4 and IL-10) expression levels were both significantly increased in *Ts*-ML-EVs groups compared to the PBS group (Fig 6A and 6B). In addition, Th1-type cytokine (IL-12 and IFN-γ) expression levels were also increased in *Ts*-ML-EVs groups (Fig 6C and 6D). However, there was no significant change between PBS group and PBS+IMS1313 group. The results confirmed that *Ts*-ML-EVs could induce the mixed response of Th1 and Th2.

## Discussion

Parasite extracellular vesicle (EVs) are an important mechanism for the delivery of various functional molecules to host tissues and cells. Exploring the composition of EVs in different parasites is critical not only to understanding how they are utilized for host-parasite communication, but also because EVs are useful in diagnosis and a rich molecular source for vaccine development and the discovery of infection biomarkers [23]. In our study, we enriched *Ts*-ML-EVs by ultracentrifugation. TEM result showed that the morphology of isolated vesicles was consistent with classical parasite EVs. Moreover, we found that these vesicles expressed EVs marker protein (enolase), which confirmed that *Ts*-ML-EVs were successfully collected.

To better understand the protein composition of *Ts*-ML-EVs, we first performed proteomic analysis of *Ts*-ML-EVs by LC-MS/MS. A total of 753 proteins were identified from *Ts*-ML-EVs, involved several enzymes including serine protease, glutamate dehydrogenase and cytosol aminopeptidase. Among them, serine protease (SP) play an important role in the interaction between *T*. *spiralis* and host. SP from *T*. *spiralis* showed the immune protective effects on sodium dextran sulfate (DSS) induced colitis in C57BL/6 mice by reducing the severity of intestinal inflammation and disease index [24]. And SP induced the mix immune response of Th1/Th2 in pig and mice, exhibited partial protective effect on *T*. *spiralis* infection [25]. Moreover, Cystatin-like protein (CLP) and DNase II identified in *Ts*-ML-EVs could also induce protective immunity against *T*. *spiralis* in mice and pig respectively [26, 27]. In addition, we found that *Ts*-ML-EVs contained Myosin-4, which regulated the formation of nursing cells by promoting myoblast differentiation [28]. However, the association of these proteins with *Ts*-ML-EVs requires further confirmation.

The protein cargo packaged into EVs before release is dependent on the cell source and their activities [29]. While differences in EVs protein content between different species may be due to their residence in host, they may also reflect different conditions during release [30]. A selective packaging of proteins in *Ts*-ML-EVs was supported by the observed subcellular localization of *Ts*-ML-EVs proteins. The proteins enriched in *Ts*-ML-EVs were mainly associated with cytoplasm and extracellular. This result may reflect the biogenetic process of *Ts*-ML-EVs [31]. This analysis of the *Ts*-ML-EVs proteome is crucial to understanding the roles of proteins in the different subcellular compartments.

We analyzed *Ts*-ML-EVs proteins from the perspective of Gene Ontology (GO) annotations, including “Biological process”, “Cellular component”, and “Molecular function”. In the “Biological process” category, we found that proteins were significant enriched in protein deglutathionylation, including protein disulphide isomerase (PDI). PDI is associated with the formation of nurse cells in host muscle cells infected with *T*. *spiralis*, thus may be important for the long-term parasitism of parasites [32]. In addition, PDI, as a DNA vaccine, can induce Th1 and Th17 dependent immune responses, and has a significant preventive effect against *Leishmania donovani* infection [33]. Whether PDI detected in *Ts*-ML-EVs in this study is a promising vaccine candidate for the prevention of *T*. *spiralis* needs further experimental verification. In “Cell component”, *Ts*-ML-EVs proteins were significant enriched in cytosolic large ribosomal subunit, including 60S ribosomal protein. In malaria, 60S ribosomal protein L6 could be a vaccine antigen provided protective immune response [34]. However, the role of 60S ribosomal protein is still unclear. “Molecular function” analysis revealed that *Ts*-ML-EVs proteins were significantly enriched in serine-type endopeptidase, including serine protease inhibitor Kazal-type 4, which belongs to a conserved superfamily, serine protease inhibitor (SPI) family. It has been reported that *T*. *spiralis* SPI could induce the alternative activation of BMDMs, which contributed to the parasitism of parasites [35]. Moreover, it has been proved that *Ts*-ML-EVs could inhibit M1 macrophage polarization to prevent colitis [19]. Our results showed that *Ts*-ML-EVs contained many functional proteins. Whether *Ts*-ML-EVs carried these proteins to regulate host immune response, translation process, and the formation of nurse cells needs further investigation.

KEGG pathways analysis revealed that *Ts*-ML-EVs proteins mainly enriched in complement and coagulation cascades, and proteasome categories. The complement system plays an important role in bridging the innate and adaptive immune system, and is especially involved in removing invading pathogens, necrotic cells, and apoptotic cells [36]. *T*. *spiralis* secreted calreticulin to inhibit the activation of classical complement in the host by binding to C1q [37]. Our study suggested that *T*. *spiralis* may also use EVs to perform this role.

Several proteasome subunits have been identified in *Ts*-ML-EVs, including proteasome subunit beta type-7 (PST) and subunits of 26S proteasome. It has been demonstrated that PST expressed at different developmental stages (intestinal infective larvae, adult worms, NBL, and ML) of *T*. *spiralis* and reduced 45.7% adult worm burden in mice [38]. Thus, PST may be used as vaccine antigen against infection of *T*. *spiralis*. HSPs play key roles in adaptation, differentiation, and protection of parasites from host killing mechanisms of hosts including reactive oxygen metabolites and low pH [39]. Our result showed that HSP83, HSP beta-1 and alpha-crystallin B chain were identified in *Ts*-ML-EVs, significantly enriched in the antigen processing and presentation, longevity regulation pathway-multiple species and protein processing in endoplasmic reticulum categories. HSP83, a number of HSP90 family, is involved in signal transduction and is a good candidate antigen of effective immunogen of leishmaniasis [40]. Alpha-crystallin B chain is a small HSP that can prevent ROS production, enhance superoxide dismutase activity, and has potential anti-cute inflammatory function [41]. Our bioinformation analysis revealed the biological and molecular functions of *Ts*-ML-EVs proteins that may contribute to the reproduction and survival of *T*. *spiralis*. Their immune regulatory functions in *T*. *spiralis* infection require further investigation.

EVs have been proposed as candidate vaccine antigens against parasite infection in animal challenge models [16]. The advantage of using EVs as vaccines is that their lipid bilayer can carry intracellular components and protect them from extracellular peptidase degradation before being transported to host cells. These components include several antigenic proteins [42]. Our present study showed that mice injected subcutaneously with *Ts*-ML-EV significantly reduced adult worms and ML burden, suggesting EVs contain antigens that can effectively reduce the transmission and pathology of *T*. *spiralis*. Antibody response is an important indicator of immune protection [43]. When parasite infection occurs, specific antibodies, acting as a strong protective immune response, prevent and inhibit parasite from attaching its host cell receptor, further helping immune cells kill and eliminate the parasite [44]. In this study, we analyzed specific antibody responses against *Ts*-ML-EVs in mice to explore the protective immune effect of *Ts*-ML-EVs. IgG antibody levels were significantly higher in the *Ts*-ML-EVs group compared to the PBS group which contributed to strong protective response of subsequent ML infection. This strong immune persisted for several weeks until the end of the experiment. Specific IgG participates in the killing and destruction of newborn larvae through ADCC. Moreover, the high levels of serum IgG and IgE might play an important role in the rapid expulsion of adult worms from the intestine of *Ts*-ML-EVs immunized mice, and in delaying the larval invasion of the enteric mucosa following challenge infection [45]. Analysis of IgE expression is important because elevated IgE antibody levels are associated with clearance of *T*. *spiralis* at the intestinal mucosal level, and mediates mast cell degranulation to impede larval invasion [46]. Most studies have shown that host infection with *T*. *spiralis* can produce Th2-based immune response, which may be related to the immune protection of *T*. *spiralis* [47]. IgG1 antibody may reflect Th2 response, and IgG2a antibody may reflect Th1 response [48]. Thus, we detected serum IgG1 and IgG2a antibody levels to elucidate the possible protective mechanisms. The results showed that *Ts*-ML-EVs induced mixed IgG1 and IgG2a antibodies responses, and IgG1 was dominant, suggesting that *Ts*-ML-EVs induced a mixed Th1/Th2 immune response. Our results provide evidence that a specific antibody response induced by *Ts*-ML-EVs is important for immune protection against *T*. *spiralis* infection.

While antibody responses are necessary for protective immunity, cell-mediated immunity is also important in eliminating parasites [22]. Our study showed that mice immunized with *Ts*-ML-EVs produced significantly higher levels of Th1 cytokines (IL-12 and IFN-γ) and Th2 cytokines (IL-4) compared to the mice immunized with PBS. It is important to note that high levels of IL-12 cytokines are essential for resistance to *T*. *spiralis*, *Neospora caninum* and *Toxoplasma gondii* infection, and blocking or lack of functioning IL-12 receptors leads to a high susceptibility to infection with parasites [49]. IFN-γ is involved in killing *T*. *spiralis* by activating macrophages and enhancing cytotoxic killing of eosinophils and granulocytes [22]. A study found that the excretion of *T*. *spiralis* is primarily regulated by CD4+/Th2 cytokines and is dependent on the production of IL-4, as inhibition of these cytokines prolongs the survival of the parasite [50]. These results indicated that *Ts*-ML-EVs can induce a mixed Th1/Th2 cellular immune response. In addition, we also measured that *Ts*-ML-EVs induced high levels of IL-10. IL-10 produced by most cells of the innate and adaptive immune systems, playing an important role in regulating immune response and protecting against *T*. *spiralis* [51]. This is consistent with our previous studies that *Ts*-ML-EVs can improve IBD by modulating innate or adaptive immune responses [18, 19].

Outbreaks of human trichinosis occur directly or indirectly due to pork consumption [52]. Therefore, the development of a vaccine against *T*. *spiralis* infection in swine would be another attractive option for disease control, especially for pigs in backyard or free-range conditions. However, most vaccine studies on *T*. *spiralis* have been carried out in mouse models, only few studies on resistance to *T*. *spiralis* infection in pigs [53]. Thus, we will future study the immune protective of *Ts*-ML-EVs in pigs in the future.

In conclusion, this study was the first proteomic analysis of *Ts*-ML-EVs, which serve as a critical source for the discovery of immunotherapeutic biologicals and parasite vaccines. In addition, vaccination of *Ts*-ML-EVs provided protective immunity against *T*. *spiralis*, highlighting their critical role in parasites infection. A better understanding of EVs will be helpful in the interaction of parasite-host and development of *T*. *spiralis* vaccines.

## Supporting information

S1 Table Top 50 enriched proteins in *Ts*-ML-EVs. (XLSX)
